# Barriers to the development of community-based nursing in Iran

**DOI:** 10.3389/fpubh.2023.1251043

**Published:** 2023-11-08

**Authors:** Hamid Peyrovi, Naiemeh Seyedfatemi, Salime Goharinezhad, Saeid Hossein Oghli

**Affiliations:** ^1^Nursing and Midwifery Care Research Center (NMCRC), School of Nursing and Midwifery, Iran University of Medical Sciences, Tehran, Iran; ^2^Preventive Medicine and Public Health Research Center, Psychosocial Health Research Institute, Iran University of Medical Sciences, Tehran, Iran

**Keywords:** nursing, community-based nursing, community health nursing, barriers, development

## Abstract

**Purpose:**

Today, the care of chronic patients and older adult people in hospitals has moved towards community-based care, and health systems focus on disease prevention, health promotion, and rehabilitation. Community-based nurses play an essential role in early identification and intervention for these conditions. On the other hand, there is an increasing trend in ageing and chronic diseases in the world especially in Iran, which increases the importance of disease prevention and public health promotion. Therefore, the current research aims to identify barriers to the development of community-based nursing in Iran.

**Methods:**

In this qualitative study, 12 semi-structured interviews were conducted with nursing experts recruited in the study by purposive sampling in 2021–2022. Interviews were recorded and transcribed and a framework analysis method was used to analyse the data.

**Results:**

The results obtained from the analysis of documents and qualitative interviews led to the identification of 4 barriers: not having a specific position, inappropriate image of nursing in society, inadequacy of education with the needs of society, and high cost of home nursing services.

**Conclusion:**

Improving the image of nursing in society, locating a special place in the health system, improving education, and adjusting the costs of home care could be the strategies that help the further development of community-based nursing (CBN).

## 1. Introduction

Health issues have changed dramatically over the past years due to ageing, old age, increase in life expectancy, and chronic diseases. Patient care in hospitals has now shifted towards community-based care, and health systems focus on disease prevention, health promotion, and rehabilitation (1). According to global statistics, non-communicable diseases are currently responsible for more than 53% of diseases in Iran (2). Also, Iran’s population is ageing like other countries in the world, and Iran’s older adult have become more older adult than the rest of the world and Asia these years, and by 2050, one out of every three Iranians will be older adult (age of 65 years old or older) (3, 4). This increasing trend has consequences on the social and economic sectors, including the resources of the healthcare sector (2). Based on this, the location and nature of nursing care have changed in response to a variety of factors and have moved from hospitals to primary care centers and social places (such as school, work, play place, etc.) (1, 5). CBN play an important role in providing primary health care (6). Nurses can provide healthcare services based on the preferences of the patient, family, and health system (7). The most important fields in which CBN can play its roles include preventive care, health promotion, education, chronic disease management, children’s and older adult health, and health policymaking (8). Many countries have established community nursing programs aligned with their healthcare systems. CBN has been significantly developed in the health systems of all countries around the world (9). For instance, in the United States, community-based nursing is an integral part of the public health infrastructure (10). In some European countries (Ireland, Sweden), CBN has replaced doctor-based and hospital-based approaches by providing health services to community members and families (11). The status of CBN in some less developed and developing countries as Indonesia, Nepal, Cameroon, Senegal, Uganda and Guyana, shows a lack of commitment and low capacity (12). In countries like Japan and China, CBN is also focusing on assessing community health needs, providing health care and health promotion (13, 14). The history of CBN in Iran dates back many years, and community-oriented curricula are taught in nursing schools, but CBN still has many obstacles and has not been able to play its role effectively in Iran’s health system (15). Now in Iran, comprehensive urban health centers are managed by graduates and associates of family health, environmental health, professional health, and CBN do not have a specific place in Iran’s health system (16, 17). Although nursing in Iran has a long history, community-based nursing still has many challenges and has not been able to play its role effectively in Iran’s health system. This study aims to analyze the state of community-based nursing to identify the challenges facing of the development of CBN in Iran.

## 2. Methods

### 2.1. Design/participant

This qualitative study was conducted through content analysis approach using purposive sampling of nursing experts in three fields and semi-structured interviews. Interviews were conducted with 12 participants at three levels of micro (nurses working in hospitals, and home care service centers), meso (faculty members of nursing schools) and macro (nurses working in the policy and legislation fields) (Table 1). Inclusion criteria included having a nursing degree, having at least one year of work experience, the ability to express verbal skills, the ability to express experiences without embarrassment, the ability to recall experiences and an innate interest in expressing experiences in the field udder study.

**Table 1:** Demographic information of participants.

Participant (no)	Sex	Working experience (years)	Department
P1	Male	20	Work in the Ministry of Health
P2	Male	18	Work in the Ministry of Health
P3	Male	15	Iranian Nursing Organization
P4	Male	15	Community Nursing Association
P5	Female	30	School of Nursing/Nursing Board
P6	Female	30	School of Nursing/Nursing Board
P7	Male	25	School of Nursing/Nursing Board
P8	Male	6	School of Nursing/Nursing Board
P9	Male	10	Home Care Nurse
P10	Male	7	Community Nurse
P11	Female	12	Community Nurse
P12	Male	15	Emergency Nurse

### 2.2. Data collection

Data were collected by the first author through semi-structured interviews with participants in 2021–2022. Every interview took between 30–45 min. The researcher explained the research goal for potential participants and took consent to record their words. The participants were asked “How do you see the state of CBN in Iran?” and “What barriers influence the development of CBN in Iran?” Probing questions were asked based on participants’ responses to the main interview questions. To ensure saturation, two more interviews were conducted and no new data were added.

### 2.3. Data analysis

Immediately after each interview, they were transcribed and reviewed to obtain a thorough understanding of the content. The transcribed interviews were analyzed based on conventional content analysis and using the framework analysis method. The five steps of framework analysis method include (1) familiarization with the interview: immersion in the data and listening and reading the interviews several times, (2) developing a working analysis: preparing a thematic framework of key topics, (3) data indexing: structuring, (4) charting: draw a diagram for each topic and transfer data, and (5) interpreting the data: explain the relationship between codes, subcategories, and categories (18). The extracted codes were classified during the reduction and condensation.

### 2.4. Data rigor

Sufficient time was spent on data collection and data analysis, and participants were selected from different groups to further enrich the data. Also, member checking was used as the interviews were read several times by the researcher and the research team, and after coding, they were checked with the participants and they were asked to express their corrective opinions about the codes. Peer review was also used to ensure the reliability of the data. In this way, the data were independently coded and categorized by the first author, and the extracted categories were analyzed. It was then sent to the research team for their additional comments. To increase the accuracy of the research in the research process, repeated bracketing has prevented the influence of one’s perspective and attitude in the research process as much as possible.

### 2.5. Ethical considerations

Data collection began after receiving the code of ethics (IR.IUMS.REC.1399.976) from the Iran University of Medical Sciences. The interviews were conducted after informing the participants of the purpose of the study and obtaining their informed consent. The participants were also assured that the data would remain confidential and anonymous, and they were assured that they could withdraw from the study at any time. A code was assigned to each of the interviewees so that they are anonymous when referring to their comments in the text (Table 1).

## 3. Results

In the present study, 12 interviews were analyzed. In the process of analyzing interviews, after removing duplicate codes and merging similar codes, 4 main categories were obtained (Table 2).

**Table 2:** Categories and subcategories.

Categories	Subcategories
Not having a well-defined position	Limiting the practice of nursing to the hospital, Limiting nursing services to hospitals, The necessity of the presence of nurses in health centers, Employment opportunities for social nursing
Inappropriate image of nursing in society	Disruption and lack of attention of the media, Presenting a different image of nursing in the media, Lack of awareness of the nurse’s role in society
Inconsistency of education with the needs of society	The need to move towards community-oriented and problem-based education, The mission of the educational system is to train a worldly, competent and efficient human force, Prioritizing nurse training based on community needs, The need to review educational programs
High cost of home nursing services	Improper pricing of home nursing services, Necessity of insurance for nursing services in the community, high price and more nursing services at home than in hospitals

## 4. Not having a well-defined position

The first main category that was obtained from the analysis of the participants’ interviews was the lack of a clear place for CBN in the Iranian health system. Not having a well-defined job position in the health system has caused CBN to not be able to perform its activities with the tasks that it is responsible for.

According to the participants in the study, nursing in Iran is limited to hospitals and medical centers, and we have fewer nurses in society. Therefore, to improve these situation, they suggested that we should recruit nurses in health centers.

“Currently, the situation of CBN is very bad, and a place for CBN in our health system has not been defined” (p1)

“Every year, we have graduates of community nursing, but most of them are working in hospitals” (p3)

## 5. Inappropriate image of nursing in society

Another challenge that was identified was the inappropriate image of nursing in society. The inappropriate image of nursing harms participant’s views and leads to less acceptance of nursing in society. Meanwhile, the media plays an important role in introducing nurses to the public. Disruption and lack of attention of the media, presenting an image of nursing that is away from the reality and is one of the most important factors affecting the social image of nursing in the society.

Lack of awareness of the role of nurses in society was one of the subcategories identified in this class. According to the interviewees, most people and officials do not have enough knowledge about the role and importance of CBN.

“Nursing is not well known in society and the media shows the face of the nurse in a way that is different from the realities of nursing. Also, the lack of people’s awareness about the knowledge of nurses has caused non-professionals to go to people’s homes in the role of nurses and lead to an inappropriate image of nursing among the people” (p11)

“Our monitoring and control mechanisms are very weak and it is not clear that someone under the title of nurse can enter people’s homes, now nurse assistants and servant are sent to people’s homes as caregivers and appear as nurses and because it creates a bad image” (p3)

## 6. Inconsistency of education with the needs of society

Another issue that was identified was the inconsistency of nursing education with the needs of the community. In this study, the participants emphasized the necessity of moving colleges towards community-oriented and problem-oriented education and training human resources based on the needs of society. In this regard, they emphasized the necessity of revising nursing education programs.

Participants also stressed this point in the interview:

“The nurses we teach in the schools work in hospitals. We pay less attention to the needs of the society. We do not have specific plans for chronic diseases and nursing services at home” (p5)

“Our society is ageing and we have not given much training to nurses to manage these cases.” (p7)

“Our nurses are not ready to face social crises.” (p8)

## 7. High cost of home nursing services

The study participants also believed that CBN plays a very important role in improving community health and empowering people.

Nurses can take care of patients at home. However, home care in Iran is very expensive and insurance still does not fully cover home nursing services.

Therefore, proper pricing of home nursing services is more important. The high cost of home care services and the lack of insurance coverage for these services have created many challenges for people to receive home care services.

“Nursing services at home are expensive, and this is the reason why people go to the hospital to receive nursing services.” (p10)

“Wealthy people in the society make the most use of home care services in Iran” (p10)

“Insurance coverage of home care services in Iran is implemented incompletely and it can even be said to be zero”? (p9)

## 8. Discussion

According to the results obtained from this study, it was found that barriers were identified for the development of CBN in Iran, which can affect its expansion. One of the identified barriers is not determining a well-defined place for CBN in Iran’s health system. CBN has been taught in Iran’s nursing schools for more than four decades, but it has not found a well-defined place until now (19). For this reason, CBN has not been able to grow and expand effectively in society. It was found in a study by Shahshahani et al. that although there are many nursing positions confirmed by the nursing authorities in Iranian hospitals and community settings, according to the statistics of the Ministry of Health, almost the majority of nurses are working in hospital departments (20), so one of the basic challenges of CBN development in Iran, the definition of job position is appropriate to be the first contact level of people with nurses in the community, and this is while the study conducted by Barasteh et al. shows that nurses are currently providing services in hospitals (21).

The next obstacle identified in this study was the high cost of home nursing care in Iran. Most insurance services in Iran do not cover nursing services at home. This budget is expensive for families and makes them go to hospitals and medical centers for services. Due to the lack of a relevant organization in Iran to regulate the costs of nursing services at home, the desired prices are usually determined based on the agreement between the client and the particular nurse. These agreements can be made based on the working hours of the nurse, the specific needs of the individual and other factors (22). Meanwhile, the insurance coverage of nursing services at home can bring more material benefits; the results of one study showed that home healthcare services can reduce treatment costs (23). In terms of cost-effectiveness, various studies show that providing community-based care through community health nurses instead of hospital care leads to improving health and quality of care, management of chronic diseases, patients’ access to community-based services, and patient satisfaction, which in turn, leads to a reduction in emergency visits and hospitalizations, and ultimately. Reduction in treatment costs (7, 24).

Another barrier identified in this study was the inappropriate image of nursing in the society. The image of nursing is tied to the identity and role of the nurse (25). One of the long-term barriers to the nursing profession is the public image of the profession. Usually, people think about people based on their appearance, and how they dress, react, communicate, or behave. They give an opinion and make a visualization or a mental image, and people interact with that organization or people based on this image, positively or negatively (26). Many reasons can cause an inappropriate image of nursing in society. In this study, the role of the media is mentioned. According to some existing studies, the image of nursing in the media does not match the reality of what nurses do professionally. In a study aimed at determining the image of nursing in the press, Sanchez Gross et al. states that what is portrayed in the press about the nursing profession is mostly a secondary role and dependent on other professions, lacking authority and lack of ability in this process (27). In a study conducted by Pawłowski et al. it was shown that The media’s expression about the nursing profession has an important effect on forming the image of this profession in society. For example, aggressive expressions from website operators about the nursing profession or even messages. The direction loaded in them by the fans of the websites leads to a negative impact on nursing in public opinion. Nurses talking about the difference between what they say and what they do can lead to the development of the nursing profession (28). Therefore, to improve the public image of the nursing profession, monitoring and taking care of the production of the media, on the one hand, and the more prominent presence of nurses in the media to introduce the reality of their profession, on the other hand, will be a helpful solution in hearing the voices of nurses correctly (29).

In this study, it was found that people’s lack of knowledge about nurses can affect the image of nursing. Accordingly, in the research conducted by Van Irsel in 2016 in the Netherlands, he emphasizes that CBNs should show themselves more outside the profession increases public awareness of the reality of the complex roles involved in this field and ensure the delivery of care (30).

Another obstacle identified in this study was the mismatch of nursing education with the needs of society. In the study conducted by Buck many experts, nursing education should be changed to acquire the skills that nurses need to meet the needs of society and the health system (31). One of the goals of the educational system for this purpose is the necessity of reforming the educational system according to the needs of society and changing the educational approach from hospital-oriented to society-oriented (7). The study conducted by Heydari et al. shows that in Iran, nurses do not get the necessary preparation to work in society at the undergraduate level (19). The establishment of a CBN education program in the form of lectures in the first months of university, introduction of textbooks, and simulations about social and home care significantly affected their attitudes and beliefs towards CBN because the traditional curriculum has made students consider social activities and home care unimportant (32). Therefore, nursing educators and planners should continually review the content of CBN education based on the needs assessment of students and graduates of community health and according to the needs of the community (33).

## 9. Conclusion

The results of this study show that there are several barriers to the development of CBN in Iran, which can be solved with appropriate measures by health policymakers and nurses. Based on this, first, a suitable place for CBN in Iran’s health system should be defined, and then nurses should receive adequate and regular training to attend these centers and society.

On the other hand, most of the costs of home nursing services must be paid by insurance companies. Also, by improving the quality of nurses’ performance and informing the public about nursing services, the social image of CBN can be improved.

### 9.1. Limitations

Considering that the sampling was purposeful and it is not possible to obtain information from all policy makers and key people, and also a number of female nurses refused to participate in the study, and on the other hand, in Iran, male nurses are more engaged in home and community care centers. Are working Therefore, the information obtained in this research may not be able to express all the facts about CBN in Iran.

## Data availability statement

The raw data supporting the conclusions of this article will be made available by the authors, without undue reservation.

## Ethics statement

The studies involving humans were approved by data collection began after receiving the code of ethics (IR.IUMS.REC.1399.976) from the Iran University of Medical Sciences. The studies were conducted in accordance with the local legislation and institutional requirements. The participants provided their written informed consent to participate in this study. Written informed consent was obtained from the individual(s) for the publication of any potentially identifiable images or data included in this article.

## Author contributions

SO, SG, HP, and NS: conception and design, critical revision of the article, and final approval of the article. SO and NS: analysis and interpretation and co-writing the article. SO: data collection and overall responsibility. All authors contributed to the article and approved the submitted version.
